# Proceedings of the Mississippi Valley Dental Association

**Published:** 1875-06

**Authors:** 


### Proceedings of Societies.

### PROCEEDINGS OF THE MISSISSIPPI VALLEY DENTAL ASSOCIATION.

WEDNESDAY EVENING, MARCH 3d, 1875.

An essay by Dr. Cushing on "Dental Education" was read by Dr. Smith.

After the reading, Dr. Watt spoke as follows: I wish to speak exactly as if the author of that paper were present. The general statement that the colleges are too lax in their examinations, may be true, but if it is true with reference to the Ohio Dental College, that institution has certainly degenerated since I left its faculty. A few years ago its examinations were the most rigid and the per cents the highest of any educational institution in America. The last time that I assisted in conducting an examination for the degree of D. D. S., the written examination occupied thirty-two hours. No student had opportunity to consult with his neighbor, his text books, or note books. The examinations were conducted in the presence of the member or members of the faculty presiding over the department of study in which they were made. There was also an oral examination of an hour in addition to this. The rule then was that in case a student's average per cent in any one department fell below eighty, he was rejected. A year or two ago, I published as a curiosity, in the "REGISTER," the questions that had been answered in writing by candidates for graduation. Not one dentist in ten to whom I talked on the subject, believed that students answered eighty per cent of such questions. Now I want an exception made, (if the Ohio Dental College has not fallen from grace,) in favor of it.

The "Dep't of the Ohio," in examining surgeons, applicants for positions in the army, were notorious all over the U. S. for the rigidity of their examination and that is the reason why nearly half the positions of responsibility in the medical department of the army were filled from Ohio, before the war closed. The written examination was six hours long and the oral examination forty minutes.

I have never been present at the examinations, of other dental colleges. But what things I have stated with reference to this dental college are facts which have gone into history.

A list of questions in anatomy was shown to a professor in one of our medical schools. He said "Do you expect students to answer those questions?" I said, "Certainly." He expressed much surprise. Yet there was not a student whose average was as low as eighty per cent, and not a student had the slightest intimation of the questions which would be asked. For my own part when I conducted the last examination I did not know myself beforehand what questions I should ask but selected them after I had made my appearance before the class.

Dr. Taft: Many can criticise and find fault and defects who could not remove those evils if they were allowed the opportunity.

Dr. Cushing speaks of the plan of appointing a board of examiners to be composed of men who are not connected with the colleges. I do not think that there exists in any of the medical schools or literary institutions of our country such a body appointed for such a purpose. It might however be well. There is no precedent to my knowledge. Why should he make this demand so imperiously, and criticise so severely for the non-observance of that which is not a custom?

There would be some advantages in the adoption of such an expedient which I can readily see. The members of the board might be expected to work with uniformity in their examinations. They would be impartial, unprejudiced, and would administer justice to all applicants for degrees.

The idea is advanced that all who go out from these institutions ought to be honest men, upright men, thoroughly prepared and as near prefection as moral training can make them. The faculty are held responsible for the very dispositions of the candidates.

If a graduate of the college conducts himself in a manner unbecoming his profession, the stigma and the odium are laid upon the faculty. Is there any justice in this?

The graduates of the medical schools do not invariably reflect honor on their teachers and their training. The same may be said of the law students. Are the colleges to be held responsible for the short-coming of their graduates?

The gentleman who wrote that essay, is I believe a member of this college association,

I do not know that this college is any better than any other college, but if it is not, it is worthy of notice that its management and guidance are in the hands of a large number of the best men in the west. They are responsible.

Dr. Cushing has done something, yes much for dental education, he has been writing and giving his influence in that direction. But if he had met with this association and here expressed his views and suggested improvements and aided in making them, and bringing about a desirable state of things, it seems to me it would have been quite as consistent as to stand at a distance and make these criticisms.

In the main I agree with him in the positions he has taken.

The members of the profession do hold us personally responsible for the improvement and elevation of the standard of professional education.

Dr. Watt: This institution had for a number of years, a board of examiners elected by the members of the association which had equal power with the faculty. I remember two instances where the faculty voted almost unaminously against giving degrees, but their decision was overruled by the board.

Dr. Berry: I think the author of the paper is about right. In England the medical student does not receive his degree from his teachers. It ought not to be permitted in America. I think he is right in what he says about the qualifications of the student. Too often the dentist receives a student into his office without any consideration of the question of his fitness for the practice of the profession of dentistry. He wants him as a menial.

If the student is not naturally acute enough to discover his preceptor's motive, so much the better pleased is the latter.

This may serve to account for the indifferent capacity of some of the matriculants of our colleges.

Dr. Osmond: I indorse that paper. The idea of having a board of examiners is good. It would have the effect of enforcing thoroughness of preparation.

Dr. Keely: I approve of his idea of concentration. There are too many colleges.

Dr. Smith: One point which Dr. Cushing makes, strikes me as particularly true of the graduates of dental colleges of the present day. Far too many show a willingness to engage in the offices of those whose practices are directly opposed to the teachings of their alma mater. From the statements of the author it would seem that comparatively few at first sight established themselves in a legitimate way and are content to wait patiently for the business which is almost sure to come. Frequently in our cities, gruduates call upon us for advice, saying they can get employment in Dr. A or B's office. He is not regarded as a reputable practitioner. What would you advise us to do? In some instances, thinking they they might do good in the capacity of missionaries, I have advised the acceptance of such situations. Usually it has been a mistake, since the result has been a thorough demoralization of the young men. It has been a query to me why so many graduates are seeking these situations. Why have they not the nerve to go out and establish themselves independently of others. It is an unfortunate characteristic of our vocation, that when a dentist is established in a legitimate practice, it is difficult for him to duplicate himself so to speak and introduce and make business for another. His individuality is so intimately associated with his business or success in practice, that the only refuge it would seem for the graduate who must be put to work by another, is in establishments where dentistry is pursued largely as a manufacturing business.

Dr. Morgan: No subject can come before this association, in which as an association and profession, we have so deep an interest as in the subject of dental education. It is so from every standpoint we may take.

Students get a wrong impression from the time they enter the office. The precepter who is in active practice has but little leisure away from his chair, to devote to the oversight of the student's studies, and the latter seeing him so occupied with the manipulative part of his duties, comes to the conclusion that a practical knowledge of details in the making of an artificial denture or the filling of a tooth, constitutes the sum total of the knowledge required to make one an accomplished dentist. Thus he comes to under rate the value of such studies as anatomy, physiology and chemistry. As a rule I would prefer that the student should enter college without the preliminary training of a preceptor.

The present organization of our schools is such that young men present themselves at the end of the second course of lectures with an expectation of securing a diploma beyond peradventure. I am inclined to think that we might adopt with advantage the university plan of holding two yearly examinations. Let it be understood that no man can graduate on any terms until he has passed his examination.

Dr. Taft: There are however some noble exceptions.

I know that the majority of those who assume to instruct students, are either not competent to the undertaking, or they neglect their duty. We all need reformation in this respect. Some can not impart to others what they themselves know. It requires a peculiar faculty to teach successfully.

Some of the best students of our colleges have never had a preliminary pupilage. One effect of an office training, as it is ordinarily conducted, is to lead the student to under rate the value of anatomy, physiology and other studies in the college course. And further than this, it frequently happens that much is learned in the office which must afterward be unlearned.

The men in the profession, who are best qualified to instruct, will not, in many cases, take students. This is unfortunate ; a change in this respect is very desirable.

A few years ago this institution, (Ohio Dental College) made a rule to the effect that a good English education should be required of those who would enter its classes. This matter gave rise to some discussion among the members of the faculty. They thought that it might operate to reduce the size of the classes. It was stricken out for that reason. I am happy to say that it has been replaced. No doubt it did operate to keep some away. I would not hesitate a moment because of the effect such an argument might have. Let us encourage young men of good natural ability and proper preparation to enter our colleges as students, and discourage others. It is far more satisfactory to teach a few who are prepared to receive instruction than a large number, most of whom are unable to understand, appreciate or value the efforts that may be made in their behalf.

We should hesitate long, before imitating the example of some of the medical colleges in this country. They make no requirements in respect to preparatory education of any kind. A young man who could not write his name, nor master the first reader in the primary school series, can enter any medical college in the country as a student, so far as I am aware, without a question. Our dental colleges are nearly at the same low level in this respect; a course of this kind is necessarily degrading.

Many of the medical colleges have reduced their fees to a mere nominal sum, thus holding out every inducement to incompetent and worthless men to enter their classes. None of our dental colleges have condescended to such a special bidding for students.

Let our aim be to discourage those whom we believe to be lacking in natural aptitude for the acquirement of the information and knowledge necessary to qualify a man for the successful performance of his responsible duties.

Dr. Rehwinkel said that he believed a pupilage in a dentist's office might be dispensed with in many cases. Too many students are from the ranks of those who have failed in other businesses. They enter an office with the view of acquiring in the shortest possible time, a sufficient knowledge of manipulations to enable them to get to practicing.

He said with regard to the feeling expressed by some that the dental profession ought to receive more fully than it does, the recognition as a specialty of medicine; that he did not sympathize with that feeling. Dentists must enter the door of the medical profession before they can expect its members to fraternize with them as their equals in standing. Thinks there is no occasion for asserting such a claim. Dentistry as a science is able to stand on its own merits. (Subject passed.)

